# Supplementary material for: External childcare and socio-behavioral development in Switzerland: Long-term relations from childhood into young adulthood
Source: PLoS One. 2022 Mar 9;17(3):e0263571. doi: 10.1371/journal.pone.0263571 (PMC8906621; doi:10.1371/journal.pone.0263571)
Supplement: S1 Table — (DOCX) [file pone.0263571.s001.docx]

Table S1. Participation and retention rates.

|  |  | **PARENTS** | | | **CHILDREN** | | | **TEACHERS** | | |
| --- | --- | --- | --- | --- | --- | --- | --- | --- | --- | --- |
| **Typical**  **Grade** | **Approx.  Age** | **Date** *(Median)* | **Participants** *(n)* | **Participation**  **Rate** *(% Target*  *Sample)* | **Date** *(Median)* | **Participants** *(n)* | **Participation**  **Rate** *(% Target*  *Sample)* | **Date** *(Median)* | **Participants** *(n)* | **Participation**  **Rate** *(% Target*  *Sample)* |
| Grade 1 | 7 | 11/10/2004 | 1240 | 74,0% | 22/03/2005 | 1361 | 81,3% | 29/03/2005 | 1350 | 80,6% |
| Grade 2 | 8 | 15/09/2005 | 1192 | 71,2% | 16/11/2005 | 1335 | 79,7% | 04/01/2006 | 1326 | 79,2% |
| Grade 3 | 9 | 13/09/2006 | 1181 | 70,5% | 15/11/2006 | 1322 | 78,9% | 27/12/2006 | 1294 | 77,3% |
| Grade 4 | 10 | n.a. | n.a. | n.a. | n.a. | n.a. | n.a. | 13/06/2008 | 1269 | 75,8% |
| Grade 5 | 11 | 30/09/2008 | 1075 | 64,2% | 02/02/2009 | 1148 | 68,5% | 07/05/2009 | 1064 | 63,5% |
| Grade 6 | 12 | n.a. | n.a. | n.a. | n.a. | n.a. | n.a. | 24/05/2010 | 977 | 58,3% |
| Grade 7 | 13 | n.a. | n.a. | n.a. | 21/06/2011 | 1366 | 81,6% | 31/08/2011 | 1269 | 75,8% |
| Grade 8 | 14 | n.a. | n.a. | n.a. | n.a. | n.a. | n.a. | n.a. | n.a. | n.a. |
| Grade 9 | 15 | n.a. | n.a. | n.a. | 27/03/2013 | 1447 | 86,4% | 24/05/2013 | 1293 | 77,2% |
| Grade 10 | 16 | n.a. | n.a. | n.a. | n.a. | n.a. | n.a. | n.a. | n.a. | n.a. |
| Grade 11 | 17 | n.a. | n.a. | n.a. | 28/03/2015 | 1306 | 78,0% | 25/05/2015 | 904 | 54,0% |
| n.a. | 20 | n.a. | n.a. | n.a. | 02/05/2018 | 1180 | 70,4% | n.a. | n.a. | n.a. |
