# Supplementary material for: External childcare and socio-behavioral development in Switzerland: Long-term relations from childhood into young adulthood
Source: PLoS One. 2022 Mar 9;17(3):e0263571. doi: 10.1371/journal.pone.0263571 (PMC8906621; doi:10.1371/journal.pone.0263571)
Supplement: S2 Table — (DOCX) [file pone.0263571.s002.docx]

Table S2. Time-points at which included outcome variables were measured.

| **Approx. age** | **7** | **8** | **9** | **10** | **11** | **12** | **13** | **15** | **17** | **20** |
| --- | --- | --- | --- | --- | --- | --- | --- | --- | --- | --- |
| **PARENT REPORTS** |  |  |  |  |  |  |  |  |  |  |
| Aggression | x | x | x |  | x |  |  |  |  |  |
| Non-aggressive externalizing | x | x | x |  | x |  |  |  |  |  |
| ADHD symptoms | x |  | x |  | x |  |  |  |  |  |
| Anxiety and depression | x |  | x |  | x |  |  |  |  |  |
| Prosocial behavior | x | x | x |  | x |  |  |  |  |  |
| **SELF REPORTS** |  |  |  |  |  |  |  |  |  |  |
| Aggression | x | x | x |  | x |  | x | x | x | x |
| Non-aggressive externalizing | x | x | x |  |  |  |  |  |  |  |
| ADHD symptoms | ^1^ |  | ^1^ |  |  |  | x | x | x | x |
| Anxiety and depression | ^1^ |  | ^1^ |  | x |  | x | x | x | x |
| Prosocial behavior | x | x | x |  | x |  | x | x | x | x |
| Delinquency |  |  |  |  |  |  | x | x | x | x |
| Delinquency, substance use |  |  |  |  | x |  | x | x | x | x |
| Substance use |  |  |  |  | x |  | x | x | x | x |
| **TEACHER REPORTS** |  |  |  |  |  |  |  |  |  |  |
| Aggression | x | x | x | x | x | x | x | x |  |  |
| Non-aggressive externalizing | x | x | x | x | x | x | x | x |  |  |
| ADHD symptoms | x | x | x | x | x | x | x | x |  |  |
| Anxiety and depression | x | x | x | x | x | x | x | x |  |  |
| Prosocial behavior | x | x | x | x | x | x | x | x |  |  |
| Delinquency, substance use |  |  |  | x | x | x | x | x |  |  |
| **OFFICIAL DATA** |  |  |  |  |  |  |  |  |  |  |
| School type (confirmed by youths) |  |  |  |  |  |  | x | x | x |  |
| Delinquency |  |  |  | x | | | | | |  |

^1^ Available; however, only two time-points were available before age 11 (i.e., before the SBQ measurement changed). With only two time-points, growth curve models with random intercept and linear slope coefficients cannot be identified (Muthen & Muthen, 2000)
