# Supplementary material for: External childcare and socio-behavioral development in Switzerland: Long-term relations from childhood into young adulthood
Source: PLoS One. 2022 Mar 9;17(3):e0263571. doi: 10.1371/journal.pone.0263571 (PMC8906621; doi:10.1371/journal.pone.0263571)
Supplement: S3 Table — (DOCX) [file pone.0263571.s003.docx]

Table S3. Internal consistency (and number of items) for the Social Behavior Questionnaire by time-point and informant (Cronbach’s alpha).

| **Approx. age** | **7** | **8** | **9** | **10** | **11** | **12** | **13** | **15** | **17** | **20** |
| --- | --- | --- | --- | --- | --- | --- | --- | --- | --- | --- |
| **PARENT REPORTS** |  |  |  |  |  |  |  |  |  |  |
| Aggression | .789 (12) | .813 (12) | .798 (12) |  | .811 (12) |  |  |  |  |  |
| Non-aggressive externalizing | .684 (9) | .742 (9) | .754 (9) |  | .760 (9) |  |  |  |  |  |
| ADHD symptoms | .794 (9) |  | .837 (9) |  | .852 (9) |  |  |  |  |  |
| Anxiety and depression | .709 (9) |  | .749 (9) |  | .787 (9) |  |  |  |  |  |
| Prosocial behavior | .766 (10) | .789 (10) | .804 (10) |  | .829 (10) |  |  |  |  |  |
| **SELF REPORTS** |  |  |  |  |  |  |  |  |  |  |
| Aggression | .716 (12) | .724 (12) | .734 (12) |  | .762 (9) |  | .839 (9) | .831 (9) | .798 (9) | .781 (9) |
| Non-aggressive externalizing | .596 (9) | .589 (9) | .612 (9) |  |  |  |  |  |  |  |
| ADHD symptoms | .582 (8) |  | .639 (8) |  |  |  | .698 (4) | .758 (4) | .778 (4) | .787 (4) |
| Anxiety and depression | .624 (9) |  | .707 (9) |  | .788 (8) |  | .825 (8) | .837 (8) | .816 (8) | .867 (8) |
| Prosocial behavior | .594 (10) | .603 (10) | .647 (10) |  | .791 (8) |  | .822 (8) | .797 (8) | .814 (8) | .790 (8) |
| **TEACHER REPORTS** |  |  |  |  |  |  |  |  |  |  |
| Aggression | .934 (11) | .934 (11) | .933 (11) | .940 (11) | .932 (11) | .937 (11) | .929 (11) | .916 (11) |  |  |
| Non-aggressive externalizing | .811 (6) | .839 (6) | .858 (6) | .835 (6) | .834 (6) | .847 (6) | .828 (6) | .848 (6) |  |  |
| ADHD symptoms | .939 (8) | .946 (8) | .945 (8) | .947 (8) | .946 (8) | .946 (8) | .945 (8) | .941 (8) |  |  |
| Anxiety and depression | .895 (7) | .909 (7) | .913 (7) | .903 (7) | .911 (7) | .918 (7) | .913 (7) | .905 (7) |  |  |
| Prosocial behavior | .922 (7) | .923 (7) | .917 (7) | .911 (7) | .915 (7) | .917 (7) | .929 (7) | .904 (7) |  |  |
