# Supplementary material for: External childcare and socio-behavioral development in Switzerland: Long-term relations from childhood into young adulthood
Source: PLoS One. 2022 Mar 9;17(3):e0263571. doi: 10.1371/journal.pone.0263571 (PMC8906621; doi:10.1371/journal.pone.0263571)
Supplement: S4 Table — (DOCX) [file pone.0263571.s004.docx]

Table S4. Descriptive statistics for the Social Behavior Questionnaire by time-point and informant (mean (standard deviation)).

| **Approx. age** | **7** | **8** | **9** | **10** | **11** | **12** | **13** | **15** | **17** | **20** |
| --- | --- | --- | --- | --- | --- | --- | --- | --- | --- | --- |
| **PARENT REPORTS** |  |  |  |  |  |  |  |  |  |  |
| Aggression | 0.601  (0.423) | 0.666  (0.442) | 0.652  (0.432) |  | 0.609  (0.431) |  |  |  |  |  |
| Non-aggressive externalizing | 0.594  (0.384) | 0.632  (0.404) | 0.588  (0.399) |  | 0.584  (0.400) |  |  |  |  |  |
| ADHD symptoms | 1.212  (0.646) |  | 1.302  (0.674) |  | 1.274  (0.690) |  |  |  |  |  |
| Anxiety and depression | 0.704  (0.464) |  | 0.854  (0.494) |  | 0.897  (0.531) |  |  |  |  |  |
| Prosocial behavior | 2.577 (0.528) | 2.685 (0.527) | 2.669  (0.532) |  | 2.708  (0.560) |  |  |  |  |  |
| **SELF REPORTS** |  |  |  |  |  |  |  |  |  |  |
| Aggression | 0.173  (0.173) | 0.141  (0.164) | 0.128  (0.160) |  | 1.543  (0.443) |  | 1.751  (0.590) | 1.689  (0.559) | 1.586  (0.484) | 1.447  (0.415) |
| Non-aggressive externalizing | 0.215  (0.185) | 0.199  (0.177) | 0.171  (0.169) |  |  |  |  |  |  |  |
| ADHD symptoms |  |  |  |  |  |  | 2.628  (0.765) | 2.702  (0.784) | 2.851  (0.809) | 2.689  (0.757) |
| Anxiety and depression |  |  |  |  | 2.039  (0.661) |  | 2.188  (0.726) | 2.333  (0.780) | 2.413  (.809) | 2.400  (.824) |
| Prosocial behavior | 0.818  (0.174) | 0.886  (0.146) | 0.911  (0.137) |  | 3.737  (0.678) |  | 3.556  (0.685) | 3.604  (0.633) | 3.737  (0.625) | 3.818  (0.549) |
| **TEACHER REPORTS** |  |  |  |  |  |  |  |  |  |  |
| Aggression | 0.588  (0.684) | 0.550  (0.638) | 0.575  (0.644) | 0.538  (0.687) | 0.485  (0.630) | 0.479  (0.637) | 0.357  (0.543) | 0.348  (0.508) |  |  |
| Non-aggressive externalizing | 0.325  (0.488) | 0.309  (0.483) | 0.334  (0.523) | 0.272  (0.465) | 0.268  (0.465) | 0.311  (0.523) | 0.233  (0.439) | 0.278  (0.485) |  |  |
| ADHD symptoms | 1.246  (0.989) | 1.102  (0.979) | 1.069  (0.953) | 1.105  (0.987) | 1.073  (0.985) | 1.001  (0.944) | 1.049  (0.942) | 1.036  (0.922) |  |  |
| Anxiety and depression | 0.871  (0.761) | 0.786  (0.732) | 0.843  (0.739) | 0.887  (0.736) | 0.899  (0.763) | 0.886  (0.773) | 0.875  (0.758) | 0.869  (0.751) |  |  |
| Prosocial behavior | 2.171  (0.824) | 2.272  (0.821) | 2.396  (0.832) | 2.201  (0.791) | 2.267  (0.834) | 2.269  (0.826) | 2.065  (0.830) | 2.064  (0.786) |  |  |
