# Supplementary material for: External childcare and socio-behavioral development in Switzerland: Long-term relations from childhood into young adulthood
Source: PLoS One. 2022 Mar 9;17(3):e0263571. doi: 10.1371/journal.pone.0263571 (PMC8906621; doi:10.1371/journal.pone.0263571)
Supplement: S5 Table — (DOCX) [file pone.0263571.s005.docx]

Table S5. Cross-informant reliabilities for the Social Behavior Questionnaire (Cronbach’s alpha).

| **Approx. age** | **7** | **8** | **9** | **10** | **11** |
| --- | --- | --- | --- | --- | --- |
| Aggression | .355 | .463 | .430 |  | .536 |
| Non-aggressive externalizing | .386 | .357 | .428 |  |  |
| ADHD symptoms | .438 |  | .469 |  |  |
| Anxiety and depression | .224 |  | .262 |  | .403 |
| Prosocial behavior | .381 | .298 | .374 |  | .512 |
