# Supplementary material for: External childcare and socio-behavioral development in Switzerland: Long-term relations from childhood into young adulthood
Source: PLoS One. 2022 Mar 9;17(3):e0263571. doi: 10.1371/journal.pone.0263571 (PMC8906621; doi:10.1371/journal.pone.0263571)
Supplement: S6 Table — (DOCX) [file pone.0263571.s006.docx]

Table S6. Bivariate correlations of types of external childcare with control variables.

|  | Family | Acquaintances | Daycare mother | Daycare center | Playgroup |
| --- | --- | --- | --- | --- | --- |
| Single parenthood | 0.01 | 0.08 | 0.08 | **0.17***** | -0.03 |
| Parental separation | 0.04 | -0.02 | 0.03 | 0.09 | 0.06 |
| Parental criminality | -0.01 | -0.02 | 0.02 | 0.03 | 0.03 |
| Parental conflict | 0.02 | -0.03 | 0.01 | 0.10 | 0.04 |
| Maternal depression | 0.02 | -0.02 | 0.01 | 0.06 | 0.02 |
| Financial problems | 0.03 | 0.02 | 0.01 | 0.01 | 0.02 |
| Maternal age at birth | **-0.17***** | -0.02 | 0.04 | 0.1 | 0.01 |
| Negative parenting | 0.06 | -0.01 | -0.05 | 0.04 | -0.02 |
| Siblings | -0.04 | -0.01 | -0.07 | **-0.22***** | 0.03 |
| Prenatal alcohol consumption | -0.01 | -0.02 | 0.00 | **0.13***** | 0.03 |
| Non-Swiss Ethnic background | -0.02 | 0.05 | 0.01 | -0.03 | -0.06 |
| Income | 0.02 | 0.00 | 0.10 | 0.05 | 0.01 |
| Parental education | **-0.11*** | -0.06 | 0.02 | **0.12**** | 0.01 |

^***^p < 0.001, ^**^p < 0.01, ^*^p < 0.05

Notes. Associations printed in bold are significant at *p* < .05.
