# Supplementary material for: External childcare and socio-behavioral development in Switzerland: Long-term relations from childhood into young adulthood
Source: PLoS One. 2022 Mar 9;17(3):e0263571. doi: 10.1371/journal.pone.0263571 (PMC8906621; doi:10.1371/journal.pone.0263571)
Supplement: S8 Table — Unstandardized coefficients from growth curve models. (DOCX) [file pone.0263571.s008.docx]

Table S8. Relations between amount of time spent in external care by acquaintances and social behavior by age. Unstandardized coefficients from growth curve models.

| Approx. age | 7 | 8 | 9 | 10 | 11 | 12 | 13 | 15 | 17 | 20 |
| --- | --- | --- | --- | --- | --- | --- | --- | --- | --- | --- |
| **PARENT REPORTS** |  |  |  |  |  |  |  |  |  |  |
| Aggression | n. s. | **b = -.04** | **b = -.05** |  | **b = -.04** |  |  |  |  |  |
| Non-aggressive externalizing | n. s. | **b = -.03** | **b =** -**.04** |  | n. s. |  |  |  |  |  |
| ADHD symptoms | b = .20,  **c = -.06** |  | n. s. |  | n. s. |  |  |  |  |  |
| Anxiety and depression | n. s. |  | n. s. |  | n. s. |  |  |  |  |  |
| Prosocial behavior | n. s. | n. s. | **b = 0.10** |  | n. s. |  |  |  |  |  |
| **SELF REPORTS** |  |  |  |  |  |  |  |  |  |  |
| Aggression | n. s. | n. s. | n. s. |  | n. s. |  | n. s. | n. s. | n. s. | n. s. |
| Non-aggressive externalizing | n. s. | n. s. | n. s. |  |  |  |  |  |  |  |
| ADHD symptoms |  |  |  |  |  |  | **b = .26** | n. s. | n. s. | n. s. |
| Anxiety and depression |  |  |  |  | n. s. |  | n. s. | n. s. | n. s. | **b = -.08** |
| Prosocial behavior | n. s. | n. s. | n. s. |  | n. s. |  | n. s. | n. s. | n. s. | n. s. |
| **TEACHER REPORTS** |  |  |  |  |  |  |  |  |  |  |
| Aggression | n. s. | n. s. | n. s. | n. s. | n. s. | n. s. | n. s. | n. s. |  |  |
| Non-aggressive externalizing | n. s. | n. s. | n. s. | n. s. | n. s. | n. s. | n. s. | n. s. |  |  |
| ADHD symptoms | n. s. | n. s. | n. s. | n. s. | n. s. | n. s. | n. s. | n. s. |  |  |
| Anxiety and depression | n. s. | n. s. | n. s. | n. s. | n. s. | **b = .07** | **b = .09** | **b = .13** |  |  |
| Prosocial behavior | n. s. | n. s. | n. s. | n. s. | n. s. | n. s. | n. s. | n. s. |  |  |

Notes. Associations printed in bold are significant at p < .05. n. s. = not significant. b = unstandardized coefficient. c = unstandardized quadratic coefficient. Gray boxes: outcome measures not available. All covariates included but not shown to avoid clutter. Coefficients displayed are unstandardized.
