# Supplementary material for: External childcare and socio-behavioral development in Switzerland: Long-term relations from childhood into young adulthood
Source: PLoS One. 2022 Mar 9;17(3):e0263571. doi: 10.1371/journal.pone.0263571 (PMC8906621; doi:10.1371/journal.pone.0263571)
Supplement: S9 Table — a. Correlations between externalizing behavior and internalizing problems at ages 0 to 1 and enrollment in external childcare at age 2. b. Correlations between externalizing behavior and internalizing problems at ages 0 to 2 and enrollment in external childcare at age 3. (DOCX) [file pone.0263571.s009.docx]

Table S9a. Correlations between externalizing behavior and internalizing problems at ages 0 to 1 and enrollment in external childcare at age 2.

|  | External childcare: Enrollment at age 2 | | | | |
| --- | --- | --- | --- | --- | --- |
|  | Family members | Acquaintances | Daycare mother | Daycare center | Playgroup |
| Aggressive behavior, age 0 to 1 | -0.01 | -0.01 | **0.11***** | 0.00 | 0.01 |
| ADHD symptoms, age 0 to 1 | 0.03 | -0.01 | -0.02 | -0.02 | 0.02 |
| Internalizing problems, age 0 to 1 | -0.02 | -0.01 | 0.01 | -0.01 | 0.00 |

^***^*p* < 0.001, ^**^*p* < 0.01, ^*^*p* < 0.05

Notes. Associations printed in bold are significant at *p* < .05.

Table S9b. Correlations between externalizing behavior and internalizing problems at ages 0 to 2 and enrollment in external childcare at age 3.

|  | External childcare: Enrollment at age 3 | | | | |
| --- | --- | --- | --- | --- | --- |
|  | Family members | Acquaintances | Daycare mother | Daycare center | Playgroup |
| Aggressive behavior, age 0 to 2 | **0.10***** | -0.01 | 0.02 | 0.04 | 0.01 |
| ADHD symptoms, age 0 to 2 | -0.02 | -0.01 | 0.03 | 0.02 | 0.00 |
| Internalizing problems, age 0 to 2 | 0.00 | -0.02 | -0.03 | 0.01 | -0.04 |

^***^*p* < 0.001, ^**^*p* < 0.01, ^*^*p* < 0.05

Notes. Associations printed in bold are significant at *p* < .05.
