# Supplementary material for: External childcare and socio-behavioral development in Switzerland: Long-term relations from childhood into young adulthood
Source: PLoS One. 2022 Mar 9;17(3):e0263571. doi: 10.1371/journal.pone.0263571 (PMC8906621; doi:10.1371/journal.pone.0263571)
Supplement: S11 Table — Unstandardized coefficients from growth curve models. (DOCX) [file pone.0263571.s011.docx]

Table S11. Relations between amount of time spent in external care by acquaintances and deviant behavior by age. Unstandardized coefficients from growth curve models.

| Approx. age | 10 | 11 | 12 | 13 | 15 | 17 | 20 |
| --- | --- | --- | --- | --- | --- | --- | --- |
| **SELF REPORTS** |  |  |  |  |  |  |  |
| Delinquency |  |  |  | n. s. | n. s. | n. s. | **b = 1.77,**  **c = - 0.69** |
| Deviance |  | **b = 0.53** |  | **b = 0.41** | n. s. | n. s. | n. s. |
| Substance Use |  |  |  | **b = 0.28** | n. s. | n. s. | n. s. |
| **TEACHER REPORTS** |  |  |  |  |  |  |  |
| Delinquency, substance use | n. s. | n. s. | n. s. | n. s. | **b = 0.50** |  |  |

Notes. Associations printed in bold are significant at p < .05. n. s. = not significant. b = unstandardized coefficient. c = unstandardized quadratic coefficient. Gray boxes: outcome measures not available. All covariates included but not shown to avoid clutter.
