# Supplementary material for: External childcare and socio-behavioral development in Switzerland: Long-term relations from childhood into young adulthood
Source: PLoS One. 2022 Mar 9;17(3):e0263571. doi: 10.1371/journal.pone.0263571 (PMC8906621; doi:10.1371/journal.pone.0263571)
Supplement: S15 Table — Unstandardized coefficients from regression models. (DOCX) [file pone.0263571.s015.docx]

Table S15. Relations between external delinquency and official measures of delinquency. Unstandardized coefficients from regression models.

|  | **Prevalence**  **Delinquency**  **(Yes/No)** | **Incidence**  **Delinquency** |
| --- | --- | --- |
| **Informant** | **Official Data** | |
| **Ages** | **10-17** | |
| Family | -0.08 | -0.07 |
| Acquaintances | -0.88 | -0.97 |
| Daycare mother | 0.02 | 0.01 |
| Daycare center | -0.09 | -0.08 |
| Playgroup | 0.04 | -0.03 |
| χ^2—^Value | - | - |
| χ^2^ df | - | - |
| CFI | - | - |
| TLI | - | - |
| RMSEA Estimate | - | - |
| SRMR | - | - |
| BIC | 26389.22 | 26876.76 |
| AIC | 25279.76 | 25762.45 |
| ^***^p < 0.001, ^**^p < 0.01, ^*^p < 0.05 |  |  |

Notes. Associations printed in bold are significant at *p* < .05. All covariates included but not shown to avoid clutter. Coefficients displayed are unstandardized.
