# Supplementary material for: External childcare and socio-behavioral development in Switzerland: Long-term relations from childhood into young adulthood
Source: PLoS One. 2022 Mar 9;17(3):e0263571. doi: 10.1371/journal.pone.0263571 (PMC8906621; doi:10.1371/journal.pone.0263571)
Supplement: S16 Table — Unstandardized coefficients from growth curve models. (DOCX) [file pone.0263571.s016.docx]

Table S16. Interaction effects between external childcare and risk on social behavior. Unstandardized coefficients from growth curve models.

|  | **Aggression** | | | | | **Non-Aggressive Externalizing** | | | | **ADHD Symptoms** | | | | **Anxiety & Depression** | | | **Prosocial Behavior** | | | | |
| --- | --- | --- | --- | --- | --- | --- | --- | --- | --- | --- | --- | --- | --- | --- | --- | --- | --- | --- | --- | --- | --- |
| **Informant** | **Self** | **Self** | | **Parent** | **Tea-cher** | **Self** | **Parent** | | **Tea-cher** | **Self** | **Parent** | **Tea-cher** | | **Self** | **Parent** | **Tea-cher** | **Self** | | **Self** | **Parent** | **Tea-**  **cher** |
| **Ages** | **7-9** | **11-20** | | **7-11** | **7-15** | **7-9** | **7-11** | | **7-15** | **13-20** | **7-11** | **7-15** | | **11-20** | **7-11** | **7-15** | **7-9** | | **11-20** | **7-11** | **7-15** |
| **Intercept** |  |  | |  |  |  |  | |  |  |  |  | |  |  |  |  | |  |  |  |
| Family*Risk | -0.00 | -0.01 | | 0.00 | 0.00 | 0.00 | 0.01 | | 0.00 | 0.02 | 0.02 | -0.03 | | -0.01 | 0.01 | -0.00 | 0.00 | | 0.01 | -0.01 | -0.01 |
| Acquaintances*Risk | 0.01 | 0.00 | | 0.00 | 0.02 | 0.00 | 0.04 | | 0.03 | 0.09 | 0.01 | -0.05 | | -0.03 | -0.04 | -0.02 | -0.01 | | 0.04 | -0.01 | -0.07 |
| Daycare mother*Risk | -0.00 | 0.00 | | -0.00 | **0.05^*^** | -0.01 | 0.00 | | 0.02 | 0.01 | -0.01 | 0.00 | | -0.03 | 0.01 | 0.01 | 0.00 | | 0.02 | 0.02 | 0.01 |
| Daycare center*Risk | 0.00 | 0.00 | | -0.00 | -0.00 | -0.00 | 0.00 | | -0.01 | -0.01 | 0.00 | -0.03 | | -0.01 | 0.01 | **0.02^*^** | 0.00 | | -0.02 | 0.00 | -0.01 |
| Playgroup*Risk | 0.00 | 0.02 | | -0.01 | 0.07 | 0.00 | -0.00 | | 0.04 | 0.01 | -0.04 | 0.01 | | -0.02 | 0.00 | 0.06 | 0.00 | | 0.00 | -0.03 | 0.04 |
| **Slope** |  |  | |  |  |  |  | |  |  |  |  | |  |  |  |  | |  |  |  |
| Family*Risk | 0.00 | 0.09 | | -0.00 | -0.12 | 0.00 | 0.00 | | -0.05 | 0.01 | -0.00 | 0.09 | | 0.08 | -0.01 | 0.02 | -0.01 | | -0.02 | 0.00 | 0.08 |
| Acquaintances*Risk | -0.01 | 0.05 | | 0.04 | **0.78^*^** | -0.02 | -0.10 | | 0.14 | -0.13 | 0.00 | 0.30 | | -0.05 | -0.01 | 0.04 | 0.01 | | 0.19 | 0.15 | **-0.70*** |
| Daycare mother*Risk | 0.00 | 0.11 | | 0.01 | -0.06 | 0.00 | 0.08 | | 0.01 | 0.02 | 0.01 | 0.08 | | 0.07 | 0.02 | -0.02 | 0.00 | | -0.21 | 0.01 | -0.03 |
| Daycare center*Risk | -0.00 | 0.09 | | 0.00 | -0.06 | 0.00 | 0.02 | | -0.07 | 0.00 | 0.01 | 0.05 | | -0.01 | 0.01 | **-0.04^*^** | 0.00 | | 0.09 | -0.04 | 0.10 |
| Playgroup*Risk | -0.01 | 0.09 | | 0.17 | -0.50 | 0.00 | **0.19^**^** | | -0.16 | **-0.21^*^** | 0.03 | -0.11 | | -0.02 | 0.02 | -0.06 | 0.00 | | -0.25 | 0.02 | -0.08 |
| **Quadratic Slope** |  |  | |  |  |  |  | |  |  |  |  | |  |  |  |  | |  |  |  |
| Family*Risk | -- | -0.28 | | -0.01 | 0.27 | -- | -0.00 | | 0.06 | -0.03 | -- | -0.12 | | -0.04 | -- | -- | -- | | 0.06 | 0.00 | -0.02 |
| Acquaintances*Risk | -- | -0.03 | | -0.00 | **-1.97^**^** | -- | 0.13 | | -0.47 | 0.10 | -- | -0.05 | | 0.06 | -- | -- | -- | | -0.91 | -0.16 | **1.92*** |
| Daycare mother*Risk | -- | -0.26 | | -0.01 | -0.23 | -- | -0.07 | | -0.20 | -0.10 | -- | -0.28 | | -0.12 | -- | -- | -- | | 0.65 | 0.00 | 0.09 |
| Daycare center*Risk | -- | -0.31 | | -0.01 | 0.09 | -- | -0.02 | | 0.17 | 0.00 | -- | -0.03 | | 0.04 | -- | -- | -- | | -0.09 | 0.04 | -0.26 |
| Playgroup*Risk | -- | -0.51 | | **-0.16^**^** | 1.14 | -- | **-0.21^***^** | | 0.48 | 0.14 | -- | 0.57 | | 0.00 | -- | -- | -- | | 0.69 | -0.01 | -0.49 |
| **Cubic Slope** |  |  | |  |  |  |  | |  |  |  |  | |  |  |  |  | |  |  |  |
| Family*Risk | -- | 0.21 | | -- | -0.16 | -- | -- | | -0.02 | -- | -- | 0.05 | | -- | -- | -- | -- | | -0.05 | -- | -0.07 |
| Acquaintances*Risk | -- | -0.01 | | -- | **1.11^*^** | -- | -- | | 0.26 | -- | -- | -0.18 | | -- | -- | -- | -- | | 0.64 | -- | -1.02 |
| Daycare mother*Risk | -- | 0.16 | | -- | 0.22 | -- | -- | | 0.16 | -- | -- | 0.15 | | -- | -- | -- | -- | | -0.46 | -- | -0.10 |
| Daycare center*Risk | -- | 0.22 | | -- | -0.04 | -- | -- | | -0.10 | -- | -- | -0.00 | | -- | -- | -- | -- | | 0.03 | -- | 0.17 |
| Playgroup*Risk | -- | 0.39 | | -- | -0.70 | -- | -- | | -0.35 | -- | -- | -0.44 | | -- | -- | -- | -- | | -0.42 | -- | 0.63 |
| χ^2^-Value | 33.07 | 51.70 | | 45.48 | 155.81 | 36.00 | 43.34 | | 115.28 | 54.38 | 48.95 | 126.87 | | 38.22 | 46.35 | 127.73 | 28.91 | | 61.61 | 65.51 | 212.08 |
| χ^2^ df | 14 | 18 | | 17 | 76 | 14 | 14 | | 72 | 17 | 14 | 72 | | 32 | 14 | 103 | 25 | | 30 | 30 | 124 |
| CFI | 0.97 | 0.98 | | 0.99 | 0.97 | 0.97 | 0.99 | | 0.98 | 0.97 | 0.98 | 0.99 | | 1.00 | 0.96 | 0.99 | 0.99 | | 0.98 | 0.98 | 0.97 |
| TLI | 0.92 | 0.90 | | 0.95 | 0.95 | 0.92 | 0.95 | | 0.96 | 0.89 | 0.93 | 0.98 | | 0.99 | 0.89 | 0.98 | 0.98 | | 0.91 | 0.94 | 0.95 |
| RMSEA Estimate | 0.03 | 0.04 | | 0.04 | 0.03 | 0.04 | 0.04 | | 0.02 | 0.04 | 0.04 | 0.02 | | 0.01 | 0.04 | 0.01 | 0.01 | | 0.03 | 0.03 | 0.02 |
| SRMR | 0.01 | 0.01 | | 0.01 | 0.02 | 0.01 | 0.01 | | 0.02 | 0.02 | 0.01 | 0.01 | | 0.01 | 0.01 | 0.02 | 0.01 | | 0.01 | 0.01 | 0.01 |
| BIC | 31058.96 | 40317.67 | | 37433.19 | 46951.79 | 31393.85 | 36593.06 | | 43282.06 | 43028.41 | 39937.56 | 52771.73 | | 44280.87 | 38393.50 | 51122.80 | 48166.44 | | 60563.74 | 61073.46 | 69984.93 |
| AIC | 30353.68 | 39443.74 | | 36651.25 | 45934.76 | 30688.58 | 35795.79 | | 42362.14 | 42246.47 | 39232.28 | 51851.80 | | 43478.49 | 37688.23 | 50361.31 | 46076.16 | | 58187.26 | 58696.99 | 67404.02 |
| ^***^p < 0.001, ^**^p < 0.01, ^*^p < 0.05 | | |  | | | | |  | | | | |  | | | | |  | | | |

Notes. Associations printed in bold are significant at *p* < .05. All covariates included but not shown to avoid clutter. Coefficients displayed are unstandardized. “*” indicates an interaction term.
