# Supplementary material for: External childcare and socio-behavioral development in Switzerland: Long-term relations from childhood into young adulthood
Source: PLoS One. 2022 Mar 9;17(3):e0263571. doi: 10.1371/journal.pone.0263571 (PMC8906621; doi:10.1371/journal.pone.0263571)
Supplement: S20 Table — Unstandardized coefficients from growth curve models. (DOCX) [file pone.0263571.s020.docx]

Table S20. Interaction effects between external childcare in a daycare center and risk on social behavior by age and informant. Unstandardized coefficients from growth curve models.

| Approx. age | 7 | 8 | 9 | 10 | 11 | 12 | 13 | 15 | 17 | 20 |
| --- | --- | --- | --- | --- | --- | --- | --- | --- | --- | --- |
| **PARENT REPORTS** |  |  |  |  |  |  |  |  |  |  |
| Aggression*Risk | n. s. | n. s. | n. s. |  | n. s. |  |  |  |  |  |
| Non-aggressive externalizing*Risk | n. s. | n. s. | n. s. |  | n. s. |  |  |  |  |  |
| ADHD symptoms*Risk | n. s. |  | n. s. |  | n. s. |  |  |  |  |  |
| Anxiety and depression*Risk | n. s. |  | **b = 0.01** |  | **b = 0.02** |  |  |  |  |  |
| Prosocial behavior*Risk | n. s. | n. s. | n. s. |  | n. s. |  |  |  |  |  |
| **SELF REPORTS** |  |  |  |  |  |  |  |  |  |  |
| Aggression*Risk | n. s. | n. s. | n. s. |  | n. s. |  | n. s. | n. s. | n. s. | n. s. |
| Non-aggressive externalizing*Risk | n. s. | n. s. | n. s. |  |  |  |  |  |  |  |
| ADHD symptoms*Risk |  |  |  |  |  |  | n. s. | n. s. | n. s. | n. s. |
| Anxiety and depression*Risk |  |  |  |  | n. s. |  | n. s. | n. s. | n. s. | n. s. |
| Prosocial behavior*Risk | n. s. | n. s. | n. s. |  | n. s. |  | n. s. | n. s. | n. s. | n. s. |
| **TEACHER REPORTS** |  |  |  |  |  |  |  |  |  |  |
| Aggression*Risk | n. s. | n. s. | n. s. | **b = -0.02** | **b = -0.02** | **b = -0.02** | **b = -0.01** | n. s. |  |  |
| Non-aggressive externalizing*Risk | n. s. | n. s. | **b = -0.01** | **b = -0.01** | **b = -0.01** | n. s. | n. s. | n. s. |  |  |
| ADHD symptoms*Risk | n. s. | n. s. | n. s. | n. s. | n. s. | n. s. | n. s. | n. s. |  |  |
| Anxiety and depression*Risk | **b = 0.02** | n. s. | n. s. | n. s. | n. s. | n. s. | n. s. | n. s. |  |  |
| Prosocial behavior*Risk | n. s. | n. s. | n. s. | n. s. | n. s. | n. s. | n. s. | n. s. |  |  |

Notes. Associations printed in bold are significant at p < .05. n. s. = not significant. b = unstandardized coefficient. Gray boxes: outcome measures not available. All covariates included but not shown to avoid clutter. “*” indicates an interaction term.
