# Supplementary material for: External childcare and socio-behavioral development in Switzerland: Long-term relations from childhood into young adulthood
Source: PLoS One. 2022 Mar 9;17(3):e0263571. doi: 10.1371/journal.pone.0263571 (PMC8906621; doi:10.1371/journal.pone.0263571)
Supplement: S22 Table — Unstandardized coefficients from growth curve models. (DOCX) [file pone.0263571.s022.docx]

Table S22. Interaction effects between external childcare and risk on delinquency and substance use. Unstandardized coefficients from growth curve models.

|  | **Delinquency** | **Deviance** | **Substance Use** |
| --- | --- | --- | --- |
| **Informant** | **Self** | | |
| **Ages** | **13-20** | **11-20** | **13-20** |
| **Intercept** |  |  |  |
| Family*Risk | -0.10 | -0.03 | 0.01 |
| Acquaintances*Risk | -0.17 | -0.38 | -0.20 |
| Daycare mother*Risk | 0.03 | -0.07 | -0.02 |
| Daycare center*Risk | 0.10 | 0.03 | **0.05^*^** |
| Playgroup*Risk | -0.15 | 0.09 | 0.10 |
| **Slope** |  |  |  |
| Family*Risk | -0.11 | 0.02 | 0.06 |
| Acquaintances*Risk | **-1.59*** | 0.38 | 0.66^*^ |
| Daycare mother*Risk | -0.36 | 0.13 | -0.05 |
| Daycare center*Risk | -0.07 | 0.04 | 0.06 |
| Playgroup*Risk | -0.65 | -0.19 | -0.15 |
| **Quadratic Slope** |  |  |  |
| Family*Risk | 0.31 | -- | -0.08 |
| Acquaintances*Risk | 1.07 | -- | **-0.49^*^** |
| Daycare mother*Risk | 0.37 | -- | 0.06 |
| Daycare center*Risk | 0.03 | -- | -0.07 |
| Playgroup*Risk | 0.77 | -- | 0.07 |
| χ^2^-Value | - | - | 21.51 |
| χ^2^ df | - | - | 15 |
| CFI | - | - | 1.00 |
| TLI | - | - | 0.98 |
| RMSEA Estimate | - | - | 0.02 |
| SRMR | - | - | 0.01 |
| BIC | 45596.03 | 58059.85 | 51299.61 |
| AIC | 44798.76 | 57344.36 | 50410.35 |
| ^***^p < 0.001, ^**^p < 0.01, ^*^p < 0.05 | | | |

Notes. Associations printed in bold are significant at *p* < .05. Due to the complexity of the calculations, the analysis for teacher-reported delinquency and substance use did not converge and is therefore not displayed. Note: All covariates included but not shown to avoid clutter. Coefficients displayed are unstandardized. “*” indicates an interaction term.
