# Supplementary material for: External childcare and socio-behavioral development in Switzerland: Long-term relations from childhood into young adulthood
Source: PLoS One. 2022 Mar 9;17(3):e0263571. doi: 10.1371/journal.pone.0263571 (PMC8906621; doi:10.1371/journal.pone.0263571)
Supplement: S23 Table — Unstandardized coefficients from regression models. (DOCX) [file pone.0263571.s023.docx]

Table S23. Interaction effects between external childcare and risk on the official delinquency data. Unstandardized coefficients from regression models.

|  | **Prevalence**  **Delinquency**  **(Yes/No)** | **Incidence**  **Delinquency** |
| --- | --- | --- |
| **Informant** | **Official Data** | |
| **Ages** | **10-17** | |
| Family*Risk | -0.03 | -0.03 |
| Acquaintances*Risk | -0.19 | -0.22 |
| Daycare mother*Risk | -0.12 | -0.09 |
| Daycare center*Risk | 0.05 | 0.06 |
| Playgroup*Risk | 0.25 | **0.22^*^** |
| χ^2^-Value | - | - |
| χ^2^ df | - | - |
| CFI | - | - |
| TLI | - | - |
| RMSEA Estimate | - | - |
| SRMR | - | - |
| BIC | 26728.58 | 27216.08 |
| AIC | 26156.89 | 26639.54 |
| ^***^p < 0.001, ^**^p < 0.01, ^*^p < 0.05 | | |

Notes. Associations printed in bold are significant at *p* < .05. All covariates included but not shown to avoid clutter. Coefficients displayed are unstandardized. “*” indicates an interaction term.
