# Supplementary material for: External childcare and socio-behavioral development in Switzerland: Long-term relations from childhood into young adulthood
Source: PLoS One. 2022 Mar 9;17(3):e0263571. doi: 10.1371/journal.pone.0263571 (PMC8906621; doi:10.1371/journal.pone.0263571)
Supplement: S1 Appendix — (DOCX) [file pone.0263571.s024.docx]

**S1 APPENDIX**

**Multiple Testing**

Due to the large number of outcome variables included in our analyses, we considered adjustments for multiple tests, such as Bonferroni adjustments. Such adjustments can be used to reduce the likelihood that results are reported that were found by chance, i.e., because a large number of tests will inevitably yield at least some statistically significant results simply because that is what chance predicts. We ultimately decided against using such adjustments because they are concerned with the hypothesis that the null hypothesis is true for all tests (i.e., testing the null hypothesis that none of the outcomes is associated with external childcare), which was not the interest of the current study. In addition, such adjustments imply that findings would be interpreted differently when fewer or more tests were performed. Furthermore, they dramatically increase the likelihood that important differences are removed due to an increased likelihood of type II errors (Morgan, 2007; Nakagawa, 2004; Perneger, 1998).

An important reason for the large number of outcome variables was that our study included many repeated measures across the participants’ life course. Prior longitudinal studies on external childcare have naturally reported outcomes on separate ages in separate publications as the study progressed, which makes the need to adjustments for multiple tests unnecessary. Using adjustments in our analysis would therefore make comparisons with other studies challenging if not impossible. Furthermore and as a statistical argument, given the longitudinal nature of our data and interrelations between different types of social behavior (Murray et al., 2020), the multiple tests were not independent in our case.

Instead of using adjustments for multiple tests, we guarded against over-interpretation of isolated findings by interpreting our results cautiously, examining overall and consistent patterns in the results rather than isolated findings.

**Residual covariances**

In the city of study, children typically have the same teacher between ages 7 and 9 and between 10 and 12. A prior analysis that examined the potential effects of teacher changes between ages 9 and 10 on teachers’ SBQ scores in the data suggested excess correlations between the data collected between ages 7 and 9 and between 10 and 12 (Murray et al., 2017). In line with prior analyses (Murray et al. 2016, 2017), we therefore included residual covariances between the scores across ages 7 to 9 and between ages 10 to 12, which improved model fit for the analyses on the teacher-reported measures.

**Other analytic considerations**

For the regressions, all predictors were centered to reduce nonessential collinearity due to scaling for predictors that were included in interaction terms and polynomials (Dalal & Zickar, 2012).

**Attrition and Handling Missing Data**

Missing data were handled as follows. First, for some quarters in the EHCs of 94 children (7.7%), interviewers did not record the number of days on which the child received external care, even though they did record that the child received external care in these quarters. In total, this affected less than 0.76% of quarters. Respondents for whom at least one quarter was missing did not significantly differ from other children on most of the control variables, except that their parents reported less maternal depression and lower income, and that they were more often from a migrant background. Further analysis revealed that this was likely due to an interviewer effect. Missing values were more prevalent for some interviewers than for others. Interviewers were allocated to respondents based on language, which, in turn, correlates with ethnicity, income, and depression (e.g., Karasz, 2005; Kleinman, 2004). In order to be able to compute overall measures for external childcare, we imputed these missing quarters with the mean of the subgroup of children who also received childcare during that quarter.

Second, as in any longitudinal study, missing data and attrition affected part of the sample. An analysis on missing data patterns in the data found that attrition was higher than average for some immigrant background groups (Eisner et al., 2019). Prior analyses on other datasets have found that the treatment of missing data can affect results in analyses on external childcare by both removing and revealing significant associations (Côté et al., 2013; Zachrisson et al., 2013). We therefore handled missing data through robust full information maximum likelihood, which has been found to be superior to other ways of handling missing data (Enders, 2001; Enders & Bandalos, 2001; Larsen, 2011).
