# Supplementary material for: External childcare and socio-behavioral development in Switzerland: Long-term relations from childhood into young adulthood
Source: PLoS One. 2022 Mar 9;17(3):e0263571. doi: 10.1371/journal.pone.0263571 (PMC8906621; doi:10.1371/journal.pone.0263571)
Supplement: S2 Appendix — (DOCX) [file pone.0263571.s025.docx]

**S2 APPENDIX**

**Do parents enroll their children into external childcare *because* of their children’s behavior?**

One relevant question for our study was whether it is possible that children’s externalizing behavior and internalizing problems started *before* they visited external childcare instead of after. In other words, did parents enroll their children into external childcare *because* of their children’s behavior? If that were the case, any increased problem behavior would not be due to external childcare per se but to the higher likelihood that children who display problem behavior are more likely to be enrolled in external childcare than other children.

Prior research has suggested that the primary reason why parents enroll their children into external childcare is not their children’s problem behavior but parental work (Baumeister et al., 2014). In the current study, we did not ask parents why they enrolled their children into external childcare and therefore could not determine whether problem behavior contributed to parents’ decision on whether or not to use external childcare services. However, the data did to some extent allow us to examine whether children displayed externalizing problems and internalizing behavior before they enrolled in external childcare.

At the end of the EHC, which was also used to obtain information about external childcare, questions about problem behavior were included. More specifically, parents were asked about longer periods during which their child showed particular problems across the first seven years of their life in three areas: aggressive behavior (longer periods during which the child was particularly aggressive and disobedient, often had outbreaks of anger, or fought with other children), ADHD symptoms (longer periods during which the child was constantly restless, fidgeting, or unable to concentrate or occupy oneself for a longer amount of time), or internalizing problems (longer periods during which the child was constantly particularly sad or anxious, withdrawn towards other children, or could not sleep because of fear).

Although these data certainly have limitations, such as being affected by memory issues, their accuracy was maximized, because the EHC was especially designed to recollect and place all events in the context of other events (such as external childcare, parental separation, etc.). For example, in this way, parents could relate their children’s externalizing behavior and internalizing problems to their enrollment in external childcare.

There is some evidence that the EHC method to retrieve information on early externalizing and internalizing behavior worked at least in part: a prior analysis on the data showed that children’s aggressive behavior and internalizing problems as recorded on the EHC were related to other events that were also recorded on the EHC (i.e., maternal depression, financial difficulties, family conflict, and parental separation) (Averdijk et al., 2012). According to the EHC, across ages 0 to 2, 2.7% of children displayed longer periods of particularly aggressive behavior, 2.4% of children displayed longer periods of continuous ADHD symptoms, and 4.7% of children displayed longer periods of continuous internalizing problems.

In order to examine how likely it is that parents enrolled their children in external childcare because of their children’s externalizing behavior and internalizing problems, we proceeded as follows. First, we examined whether or not children who were enrolled in external childcare had displayed externalizing behavior and internalizing problems before they started external childcare. Then we examined whether these rates of externalizing behavior and internalizing problems were related to the likelihood that children enrolled in external childcare. We note that we did not examine children who enrolled into external childcare at ages 0 to 1 in these analyses, because we had no or few data on their externalizing and internalizing problems before this age. This is a limitation of our analyses. We did examine the relation between externalizing behavior and internalizing problems in the first year of life and enrollment in external childcare at age 1 and found no statistically significant results. However, the prevalence of externalizing behavior and internalizing problems in the first year of life was very low (less than 2%).

The results of our analyses are shown in Table S9a and S9b. Overall, the vast majority of relations was not statistically significant, suggesting little evidence that children who displayed externalizing behavior or internalizing problems were more likely to be enrolled into external childcare later on compared to other children. Only two statistically significant results emerged. First, children who displayed aggressive behavior in the first two years of life were more likely to start visiting external childcare by a daycare mother at age 2 (see Table S9a). This relation was no longer statistically significant one year later, however, as shown in Table S9b. What is more, it applied to only very few children, as only 2% of children started visiting a daycare mother at age 2, whereas most children who visited a daycare mother started doing so at an earlier age.

The second statistically significant result was that children who displayed aggressive behavior at ages 0 to 2 had a higher likelihood of starting external childcare by family members at age 3. This suggests that children’s aggressive behavior may have played a role in their parents’ decision to start using external childcare by family members. It is noted, however, that only 2% of children started attending external childcare by family members at age 3, whereas the majority of children who attended external childcare by family members started doing so at an earlier age. What is more, we found no relation between external childcare by family members and externalizing behavior in the main part of the current paper. Thus, if anything, for a small group of children, external childcare by family members may have decreased children’s problem behavior.
