# Supplementary material for: External childcare and socio-behavioral development in Switzerland: Long-term relations from childhood into young adulthood
Source: PLoS One. 2022 Mar 9;17(3):e0263571. doi: 10.1371/journal.pone.0263571 (PMC8906621; doi:10.1371/journal.pone.0263571)
Supplement: S3 Appendix — (DOCX) [file pone.0263571.s026.docx]

**S3 APPENDIX**

The type of external childcare that children attended was to some extent associated with characteristics of their family. Table S6 displays these associations. Although we found no significant relations of the family characteristics with childcare by neighbors and acquaintances, daycare mothers, and playgroups, there were relations for external care by family members and daycare centers. Children who had relatively young mothers and children whose parents had a relatively low level of education were more likely to spend time in family-based childcare. Children from single-parent households, children who had no or few siblings, and children whose parents had a relatively high level of education were more likely to spend time in daycare centers. In addition, children from more vulnerable backgrounds (e.g., prenatal alcohol consumption) were more likely to spent time in a daycare center.
