# Supplementary material for: External childcare and socio-behavioral development in Switzerland: Long-term relations from childhood into young adulthood
Source: PLoS One. 2022 Mar 9;17(3):e0263571. doi: 10.1371/journal.pone.0263571 (PMC8906621; doi:10.1371/journal.pone.0263571)
Supplement: Attachment — Submitted filename: Response to Reviewers.docx [file pone.0263571.s027.docx]

**Responses to Reviewers**

**Reviewer 1**

1. The introduction section could benefit from a more comprehensive review of the most recent literature on the topic. Given that this article seeks to address a research question that (1) has been very controversial in the field of human development (Dearing and Zachrisson, 2017), and (2) has been most recently approached through quasi-experimental approaches to overcome long-discussed methodological issues of endogeneity/selection bias, I suggest the authors should not only focus on presenting correlational studies but should be more exhaustive in presenting recent research that has used quasi-experimental approaches and strategies to address issues of confounding. Some examples of such studies include:
   - Jaffee, S. R., Van Hulle, C., & Rodgers, J. L. (2011). Effects of nonmaternal care in the first 3 years on children’s academic skills and behavioral functioning in childhood and early adolescence: A sibling comparison study. Child Development, 82, 1076–1091. doi:10. 1111/j.1467-8624.2011.01611.x
   - Crosby, D. A., Dowsett, C. J., Gennetian, L. A., & Huston, A. C. (2010). A tale of two methods: Comparing regression and instrumental variables estimates of the effects of preschool child care type on the subsequent externalizing behavior of children in low-income families. Developmental Psychology, 46, 1030–1048. doi:10.1037/a0020384
   - Baker, M., Gruber, J., & Milligan, K. (2019). The Long-Run Impacts of a Universal Child Care Program. American Economic Journal. Economic Policy, 11(3), 1-26. <https://doi.org/10.1257/pol.20170603>

   Our response: We have added a discussion of studies that have gone beyond regular/OLS regressions with covariate adjustment, including sibling comparisons, propensity score matching, instrumental variables, and policy change studies. The discussion of these studies is now included on pp. 7 and 8.
2. Moreover, the authors should also include recent studies that have called into question the internal validity of correlational studies on the relation between time spent in childcare and behavior problems. This recent research has found that conflicting findings on the subject are mostly due to model specifications and the degree of rigor in eliminating selection bias in the models. Indeed, when more rigorous statistical designs have been used to rule out selection bias, the relation between time in child care and behavior problems is, in some studies, no longer statistically significant, suggesting issues of confounding in correlational evidence. Please see the following references for a review of studies:
   - Dearing, E., & Zachrisson, H. D. (2017). Concern Over Internal, External, and Incidence Validity in Studies of Child-Care Quantity and Externalizing Behavior Problems. Child Development Perspectives, 11(2), 133–138. https://doi.org/10.1111/cdep.12224
   This is also the case for studies that have looked at the association between early child care and behavior problems during adolescence. When studies have used approaches that are less rigorous in ruling out selection bias (using only covariate-adjustment), they have found positive associations between time spent in child care and externalizing behavior problems (e.g., Vandell, Burchinal, and Pierce, 2016), whereas studies using some strategy to address selection, for instance reducing imbalance in observed characteristics by using propensity score matching (e.g., Orri et al. 2019) the relation between child care and behavior problems is even negative. Nonetheless, these important problems related to internal validity are not mentioned in the literature review.

   Our response: We have included a section on concerns about causality and validity in our manuscript. The studies by Vandell et al. (2016) and Orri et al. (2019) were mentioned as references in the previous version of the manuscript but we agree with the reviewer that we did not extensively discuss the broader issue of selection bias and methodological concerns. To be clearer with regards to these issues, we have added an extensive section starting at the end of p. 6 through p. 8 on validity issues and studies using alternative methods in the larger context of questions of causality. We have attempted to provide a balanced discussion, giving space to both reviews that have concluded that there is little support for causality and also to reviews that have concluded that consider causality to be mostly supported.
3. Considering that reviews of the literature clearly demonstrate these issues of selection, I suggest authors revise some of their claims, such as “Our findings on externalizing behavior are largely in line with the international studies discussed earlier, the majority of which have found that more time spent in a daycare center is related to increased externalizing problems at earlier ages (lines 509-511)”. It is not true that the majority of the international evidence indicates such a relation, and most importantly, even if this was the case, the important issue is not whether the majority of papers show a positive association, as if these studies were poorly conducted any inference would be invalid. Instead, the focus should be on internally-valid studies that have used approaches that go beyond traditional covariate adjustment to mitigate issues of selection bias. As already mentioned, such studies tend to find no relation at all and even reductions in behavior problems as children spend more time in child care.

Our response: We have revised parts of our claims so that the mixed nature of prior research is clearer. We aimed to provide a balanced overview of the international literature by adding a paragraph (bottom of p. 4, top of page 5) where we discuss the conclusions of past literature reviews (Belsky, 2001; Huston et al., 2015; Jacob, 2009; see the first page of Dearing & Zachrisson, 2017 for their summary of past reviews) while at the same time highlighting the shortcomings of prior studies, namely that there are inconsistencies and open questions.

4. Furthermore, I suggest the authors to revise whether some of the references cited in the article might be equivocally used. For instance, McCartney’s et al. (2010) mentioned that “the evidence linking child care hours with externalizing behavior was equivocal in that results varied across model specifications” (McCartney et al., 2010), and for such reason they tested five causal propositions that “if satisfied, would be consistent with the view that child care hours and externalizing behavior are causally linked.”, which they then show are not satisfied. Since one of the initial claims made in the introduction is that “Prior research on the amount of external childcare has generally concluded that more time in external childcare is related to increased problem behavior.”, citing McCartney’s (2010) article, it seems inaccurate.

   Our response: We have moved the reference to McCartney article to the part where we discuss different methods yielding different results (p. 7).
5. Regarding the External childcare in Switzerland subsection, I suggest the authors give a more comprehensive review of the ECEC policies/contexts in Switzerland, and findings are also contextualized accordingly. Switzerland had (still has) the most expensive ECEC in the world (about 50% of disposable income, on average, goes into paying for ECEC), and the country has a very family/mother-unfriendly policies (see Chzhen, Gromada, & Rees, 2019, Are the world’s richest countries family friendly? Policy in the OECD and EU). Although the authors put attention to the counterfactual modes of care, they run the risk of overemphasizing results from a very uncommon context if it is not addressed in more detail.

   Our response: We have added more context by placing Switzerland’s policies for early external care in a more international context. We have tried to provide a nuanced picture since Switzerland is rated poorly on some indicators (at least compared to other European countries, though not necessarily to other countries) but ranks higher or average on others (pp. 11-12).
6. I was disappointed that the authors (from the introduction to the discussion) paid little attention to questions of causality and selection, which are central to the research questions being addressed. Although the authors mention how this study cannot draw causal relations because it uses observational data (lines 325,326), I suggest they clarify that it is not only because of the type of data, but also because the statistical design used does not allow the authors to credibly discard the potential confounders. Even if authors had been capable to rule out reverse causality successfully and prove that parents did not enroll their children into external care due to their problem behavior, there are many other threats to validity that this study does not consider for relying exclusively on covariate-adjustment. Given the implications of a study like this could potentially have, it would not be responsible to present evidence suggesting that child care might be detrimental for young children while relying on a weak empirical strategy that is prone to bias.

   Our response: We revised our manuscript to bring attention to the issue of causality. To clarify this point early on in the manuscript, we included two short sentences on p. 4 (“In order to control for the possibility that our results are spurious and explained by third factors, we included a large number of confounding factors. Nevertheless, like other studies on the same topic, our study is observational and we therefore cannot be sure that the remaining relationships are more than correlational.”). In addition, as mentioned above, we have included an extensive discussion of causality issues in the introduction.
   We also replaced the first two points in our discussion of limitations by a longer section where we discuss the limitations regarding causality in our data and statistical design on pp. 31-32.
7. Given this, my biggest concern with the paper is the lack of rigor in the language used, as these correlational results could have great implications in practice (i.e., policies on early child care provision, female participation in the labor market). Although the authors acknowledge none of the findings are causal, through the results and discussion they seem to suggest that there might be a causal link. For instance: “Children from less vulnerable backgrounds, however, did not profit from more time in a daycare center or even fared worse” (lines 468-469), or “Though there were some results suggesting beneficial relations (e.g., the association between external family care and prosocial behavior, and decreased externalizing behavior among vulnerable children in daycare), these were offset by less positive ones (e.g., the association between daycare and externalizing, and increased internalizing problems and substance use among vulnerable children in daycare). I encourage authors to address results with much caveat and some careful explanation.

   Our response: We have revised the sentences that the reviewer highlights while at the same time being clear about the direction of the results as follows:
   “The same was not the case for children from less vulnerable backgrounds, for whom results showed no relations or for whom more time in a daycare center was related to more externalizing behavior.” (p. 26).
   “Though there were some results suggesting relations between childcare and productive youth development (e.g., the association between external family care and prosocial behavior, and decreased externalizing behavior among vulnerable children in daycare), other results suggested relations between childcare and increased problem behavior (e.g., the association between daycare and externalizing, and increased internalizing problems and substance use among vulnerable children in daycare), highlighting the necessity of examining various outcome domains.” (p. 31)
   We have also gone through the rest of the manuscript and used the term “relations” and “related”, which we feel is more neutral, as much as possible.
8. Minor comments:

   Methods and Statistical Analyses
   Given that ‘time in external childcare’ is the predictor of interest, I suggest moving the information about the data collection using the Event History Calendar back to the article to provide more detailed description of (i) who reported the data, and (ii) the reliability of the measure. Otherwise it becomes less clear who actually reported the childcare information (whether it was the child or the parents) and how the EHC design provides accurate enough data of time spent in childcare that is collected retrospectively. Also, I suggest authors not only give evidence supporting the use of an EHC but also discuss how retrospective self-reported data can bias the results.

Our response: As recommended by the reviewer, we moved our discussion of the EHC back into the manuscript. It is now included on p. 14.
Also, we have extended our previous limitations section on memory effects to more generally bias in retrospective self-reported data and included references to prior work (p. 33).

9. It is not clear to me whether the final sample consisted of the 94% of 1,675 first graders that had participated in at least one of the data collections (line 194) or the 1,225 children whose parents had completed the EHC (line 239). I also suggest including the sample size in each of the regression models to be able to compare and check the sample sizes of each model (or clarify that they are the same given that you have done multiple imputation).

   Our response: We had previously included the final sample size in the section on External Childcare (Method section, p. 12) and Delinquency and Substance use (p. 14) which we agree was too hidden and scattered. We have now moved both sections to the Analytic Strategy section and also mention there that we used FIML for missing data on other variables to clarify our procedure (p. 19). In addition, we have included sample size in each of the tables in the main manuscript for clarification.
10. I suggest authors discuss further why some of the results showed different directions in the association at different ages: “For example, daycare center care was associated with more self-reported aggression at ages 7 and 8, but the relation flipped at ages 13 and 15, where center care was related to less aggression.”

Our response: We performed additional analyses to find out what was driving this effect. Specifically, because center-based childcare might be associated with more problem behavior but also with better educational achievement, and because problem behavior generally correlates with worse educational achievement, we suspected that the finding that more time spent in daycare centers was associated with less aggression might be due to better educational achievement and not to less aggression per se. We therefore performed additional analyses of self-reported aggression at ages 13 and 15 on external childcare where we included educational achievement as a predictor. In both cases, the association of daycare centers with self-reported aggression at ages 13 and 15 turned non-significant. Thus, the previously detected association seemed to be due to the association between daycare centers and educational achievement, and not due to decreased aggression per se.
We did not find that higher educational achievement removed other relations between external childcare and problem behavior, specifically the relation between external childcare and higher ADHD symptoms at ages 15 and 17.

We found it challenging to include these additional robustness checks in this manuscript because we suspect that it might confuse the reader to introduce these additional variables (i.e., educational achievement) and analyses and it only concerns a very small subsample of the analyses. In addition, we are currently working on a separate manuscript that focuses explicitly on the relation between childcare and educational outcomes in our data, which we could not fit into the current, already dense manuscript. Our previous version of the manuscript therefore did not include it. However, in order to answer the reviewer’s (and potential readers’) question regarding these relations, we have now added an appendix (see Online Appendix S4) with a short discussion of these follow-up analyses. We hope this answers the question.

11. There are some references that are cited in the text but are not included in the reference list. For example: Vandell et al., 2010 (line 123), Wustmann Seiler et al., 2017 (line 174).

    Our response: We thank the reviewer for this and have added the references to the reference list.

**Reviewer 2**

1. The article is well built and well written from a theoretical point of view, and provide a lot of estimations and robustness checks. The article is very (too much?) ambitious, treating several outcomes at different ages, with quite limited, non-representative sample.

Our response: Our sample is reasonably large and representative of the larger population of 7-year-olds in the city, for example the high rate of immigrants (as reported in the Method section). Over time, attrition was higher than average for some groups of immigrant backgrounds. We therefore treated missing data through robust full information maximum likelihood (please see Online Appendix S2).

2. After reading the summary and the article, I have not a take-away message since results go in several directions according to the informant and child’s age. The major weakness is that the causality between external childcare when the child was young and child’s future development or trend in child’s future development (that the model tests) are not established. How to be sure that the relation observed is causal and not a spurious relationship due to unobserved confounding variable? Even the valuable efforts of authors to control for many of them, this is not enough and I do not belief that growth curve models are particularly adequate for the topic. I would recommend the authors to use policy evaluation methods to treat better causality and to limit the ambitions of the article by limiting the number of outcomes and time periods.

   Our response: We understand the reviewer’s concern about causality. We have therefore added discussions and limitations regarding causality to several places in our manuscript. On p. 4, we have added statement that our study is observational and we cannot claim causality. A longer discussion on causality is included on pp. 6 to 8. Furthermore, we have extended our section on the limitations involved in our method and data on p. 31 and 32. We realize that the conclusions to be drawn from our study are limited and have therefore gone through the manuscript and removed or changed any sentences that might suggest causality. Unfortunately, we do not have data on policy changes and could therefore not evaluate those.
3. I wonder whether the use of growth curve model is adequate for the topic since the event we are interested in (external childcare when child is very young) occur many years before the large period of observation of outcomes (from age 7 to 20). It might affect rather the initial level rather the hereafter trend. I do not see any rationale why the daycare would affect the rate of change at which child developed after the initial level.

   Our response: We agree with the reviewer that external childcare might affect the initial level rather than later points in time, or at least that the statistical “effects” might be larger at earlier ages than later ones. For this reason, we did not only report the rate of change and the intercept at the mean but instead ran analyses at each individual age because only with these analyses, one can gain an overall picture of whether and when (i.e., at which age) the relations exist. In this way, the focus is not on one point in time but on every time point included. In other words, in this way, we can answer the question of whether it is the initial level rather than the trend where the relations exist.
4. If authors keep this method, a short explanation for non-initiated reader is anyway really needed. The authors comment the different parameters without reminding what they mean (inter-individual variability or intra-individual patterns of change over time), so the results are not easily readable for non expert.

   Our response: We have re-structured the wording on the intercept versus slope coefficient in the Analytic Strategy section to be clearer. We have also included an additional explanation to guide readers in the Results section on pp. 20-21.
5. The sample size is only mentioned once, and should be precise in each table. Do the sample size available is the same for all regressions? If not, what has been the choice to keep the common individuals interviewed each wave or to keep maximum individuals at each wave?
   The age range from 7 to 20 is very large

   Our response: We communicated our sample size more clearly (in the Method section, p. 19), and have also added sample size to all tables in the manuscript.
   The same individuals were interviews in each wave as part of the aim of the longitudinal nature of the study, which was to follow individuals’ development over time. The study does have attrition, as reported, and we therefore used FIML to handle the missing data.
   It is correct that the age range from 7 to 20 is very large. We considered splitting up the results in separate papers but preferred to report all results on the relation between early childcare and social behavior in one paper as a more straightforward way to communicate our findings.
6. I would like to see a description of selection into external childcare. Who are the parents who use external childcare and which type of childcare?

   Our response: We have added an online appendix (S3) which discusses the relation between external childcare and family characteristics.
7. Some results are weird, particularly when they differ by informant. They seem sensitive to measurement, so I have doubts about their validity.

Our response: Several studies have shown that the multi-informant SBQ data are reliable and valid, and we have added additional references that show this (p. 15).
In addition to this, we have added references showing that cross-informant reliabilities for cross-behavioral assessments are usually low and we have added an explanation for why we could not combine the scores across informants and instead estimated our analyses for each informant separately (p. 16).

8. A discussion is missing about the non representative sample. As it is a sample based on a town, it may be possible to control for the daycare attended more precisely.

   Our response: We are not entirely sure why our sample would not be representative. The sample comes from the largest city in Switzerland and is reasonably representative. As in any longitudinal study, there was attrition, which is shown in Table S1 (Online Appendix). It is correct that attrition was higher among some immigrant background groups. We handled these missing data through FIML (please see Online Appendix S1).
9. The fact that more internalizing problems and substance problems are only observed for children attending daycare coming from vulnerable background put doubts on the role of unobserved characteristics. Who are these children? Do they attend same daycare than others? Do they have the same life-course?

Our response: Unfortunately, we did not have information on the type of center care (quality, child/caregiver ratio, etc.) and so we are unable to examine whether these children attend the same daycare as others. Our analyses reported in Online Appendix S3 suggest that only few family background characteristics played a role in selection into external childcare. Single parenthood increased the likelihood of attending center care, as did a lower number of siblings, prenatal alcohol consumption, and higher parental education. Thus, some of the risk factors that were included in the vulnerability score were related to center care. Furthermore, a lower maternal age was related to family care. We did not find significant correlations between family factors or vulnerability indicators and the other types of external care.
We have not done additional analyses on how the life course of children from vulnerable backgrounds differs from those of other children. We agree that this would be interesting for future analyses.

10. More importantly, several unobserved characteristics are possible and strong mediator of results: for instance whether the mother was working part-time full-time (probably by financial necessity for children coming from disadvantaged background) part-time or not working at all, the type of kindergarten attended (of worse quality), the instability of family structure (dissolution and the arrival of a step-parent) from Kindergarden or hereafter, etc.

    Our response: We agree that the range of covariates that we included is not exhaustive and that additional factors may have played a role. As mentioned, we could unfortunately not include childcare quality because this information was not available. We did, however, control for several types of family structure (e.g., parental separation, criminality, depression, financial problems, etc, please see Online Appendix S3). We agree with the reviewer that there are possibly more third variables that may explain our results and we can therefore not claim that our findings are causal. We have added a paragraph to our limitations section (please see the first mentioned limitation, pp. 31-32) where we discuss this.
11. Minor remarks:
    Why is the rationale of precising that the sample is multi-ethnic in the summary since no much emphasis on this point is put in the article?

Our response: We have removed the term “multi-ethnic” from the abstract.

12. The limitations section should come back on data, and selection in use of external child care.

    Our response: We agree and have added a paragraph that discusses the issue of selection in our data (please see the first mentioned limitation, p. 31-32).
