# Supplementary material for: External childcare and socio-behavioral development in Switzerland: Long-term relations from childhood into young adulthood
Source: PLoS One. 2022 Mar 9;17(3):e0263571. doi: 10.1371/journal.pone.0263571 (PMC8906621; doi:10.1371/journal.pone.0263571)
Supplement: Attachment — Submitted filename: Response to Reviewers.docx [file pone.0263571.s028.docx]

**Responses to the Reviewers**

**Reviewer 1**

1. Although the authors have included the suggested literature, I’m worried that the conclusion inferred from the causal evidence and from the most recent reviews (e.g., Dearing & Zachrisson, 2017; Huston, 2015; McCartney et al., 2010) is still wanting. It is not that “different methods may yield to different results” but that more rigorous methods with better identification strategies to address selection bias lead to more internally valid results. I am afraid this idea is still not well addressed in the article and that the reader might potentially draw the wrong conclusions from the article because of this.
   This is the case in statements such as: “Past literature reviews on the link between the quantity of external childcare and child development have concluded that external childcare is related to increased problem behavior (see Dearing & Zachrisson, 2017, for an overview).”, which does not clarify that this is the case when less rigorous methodologies such as covariate-adjustment/OLS are implemented but that the opposite relation is found when selection bias is addressed through quasi-experimental approaches. Another example is: “Other studies have used other methods, which have their own unique advantages and disadvantages, such as sibling comparisons, propensity score matching, and instrumental variables and shown that different methods may yield different results (e.g., McCartney et al., 2010).”

Our response: We have removed the reference to the Dearing & Zachrisson article in the first sentence mentioned by the reviewer and have instead explicitly included worries about selection bias with a reference to the mentioned article on p. 5:

“For example, it is currently unclear to what extent study results are causal or affected by selection bias (Dearing & Zachrisson, 2017), whether conclusions extend to all or only some forms of external childcare and to different types of problem behavior, whether the link between external childcare and problem behavior represents the populations most at risk, and how the relation develops across the life course.”

On p. 6, we have also added an extra heading, “Validity and selection bias” to lead the section describing the results of studies using methods other than covariate adjustment. We hope that this makes this section more prominent.

Instead of arguing that different methods yield different results (i.e., the prior sentence stating that “Other studies have used other methods, which have their own unique advantages and disadvantages, such as sibling comparisons, propensity score matching, and instrumental variables and shown that different methods may yield different results (e.g., McCartney et al., 2010)”), we have added on p. 7 that other articles have gone beyond covariate adjustment and yield less biased results:

“Most studies (including ours) have attempted to control for omitted variable bias by controlling for a range of background characteristics, such as a family’s socio-economic status and parenting characteristics but potential bias from unmeasured characteristics remains. Other studies have gone beyond ordinary regressions using covariate adjustment, such as sibling comparisons, propensity score matching, and instrumental variables. Although each method has their own unique advantages and disadvantages and cannot match a causal experiment, these methods yield less biased results.”

In addition, we have more explicitly acknowledged the findings by Dearing and Zachrisson (2017):

“In their review of these studies, Dearing and Zachrisson (2017) concluded that in six of the studies, time in childcare predicted more problems, that one predicted the opposite, and that six found null results.”

2. I strongly suggest that the authors relate the specific methods used in the articles they mentioned to make particularly strong claims. Again, this kinds of statements that have strong policy implications must be stated with enough context. For instance, "the previously mentioned NICHD study found that even though low-quality external care is more often associated with problem behavior than high-quality care, external care was related to more externalizing behavior at all levels of quality (NICHD Early Child Care Research Network, 2003)." or "A Swiss study showed that an increased availability of external childcare provisions would affect parental labor participation only marginally (Stern et al., 2018)." What analytical strategy was this study using? What was the identification strategy used? What was the sample studied? Was it nationally-representative?

Our response: We added information about the study design and analytical strategy for the two sentences highlighted by the reviewer:

p. 31/32: “However, the previously mentioned NICHD study, which used a multisite longitudinal design in the US and regression analyses with covariate adjustment, found that even though low-quality external care is more often associated with problem behavior than high-quality care, external care was related to more externalizing behavior at all levels of quality (NICHD Early Child Care Research Network, 2003).”

p. 12: “A Swiss study among a representative sample of 1,000 Swiss participants performed a so-called “choice experiment” where participants could choose between different forms and quantities of external childcare. Results based on regression models showed that an increased availability of external childcare provisions would affect parental labor participation only marginally (Stern et al., 2018).”

We also added information on study design and/or analysis techniques to the sections on international studies on the type of childcare (p. 5), earlier ages (p. 6), the link with internalizing problems (p. 6), and prior Swiss studies (p. 12) in order to further point to the fact that most studies were based on regression-based analyses with covariate adjustment rather than quasi-experimental designs or statistical techniques.

3. Although the data used in this study is rich in that it follows children until adolescence and early adulthood and it includes multiple outcome variables, I want to reiterate that this study fails to address selection effects, making it difficult to draw meaningful conclusions. While the authors employ covariates and some initial robustness checks, controlling for observed characteristics, the growth curve model employed is insufficient to establish internally valid estimates given that potential unobserved characteristics that relate to both time spent in external child care and the outcomes might still not be accounted for. It is exactly for this reason that recent research has moved away from correlational designs to address a topic that is very sensitive in terms of policy implications for early childhood care and education supply.

Our response: We have added a paragraph to our Analysis section explaining why we did not use a quasi-experimental study design or statistical technique:

“Due to the nature of our sample and data, we could not perform robustness checks to check the sensitivity of our findings against selection bias. For example, change-on-change fixed effects models were not applicable in our case because we analyzed the relation between one prior predictor (external childcare) and a range of later outcomes. Neither could we use sibling fixed effects because with the exception of very rare cases, our data did not include siblings. In addition, propensity score matching requires the matching variables to be measured prior to the main predictor, which was not possible in our data.”
